# Application of a Combined Peptidomics and *In Silico* Approach for the Identification of Novel Dipeptidyl Peptidase-IV-Inhibitory Peptides in *In Vitro* Digested Pinto Bean Protein Extract

**DOI:** 10.3390/cimb44010011

**Published:** 2021-12-28

**Authors:** Serena Martini, Alice Cattivelli, Angela Conte, Davide Tagliazucchi

**Affiliations:** Department of Life Sciences, University of Modena and Reggio Emilia, Via Amendola, 2-Pad. Besta, 42100 Reggio Emilia, Italy; serena-martini@unimore.it (S.M.); alice.cattivelli@unimore.it (A.C.); angela.conte@unimore.it (A.C.)

**Keywords:** mass spectrometry, diabetes, bioactive peptides, screening approach, gastrointestinal digestion

## Abstract

The conventional approach in bioactive peptides discovery, which includes extensive bioassay-guided fractionation and purification processes, is tedious, time-consuming and not always successful. The recently developed bioinformatics-driven *in silico* approach is rapid and cost-effective; however, it lacks an actual physiological significance. In this study a new integrated peptidomics and *in silico* method, which combines the advantages of the conventional and *in silico* approaches by using the pool of peptides identified in a food hydrolysate as the starting point for subsequent application of selected bioinformatics tools, has been developed. Pinto bean protein extract was *in vitro* digested and peptides were identified by peptidomics. The pool of obtained peptides was screened by *in silico* analysis and structure–activity relationship modelling. Three peptides (SIPR, SAPI and FVPH) were selected as potential inhibitors of the dipeptidyl-peptidase-IV (DPP-IV) enzyme by this integrated approach. In vitro bioactivity assay showed that all three peptides were able to inhibit DPP-IV with the tetra-peptide SAPI showing the highest activity (IC50 = 57.7 μmol/L). Indeed, a new possible characteristic of peptides (i.e., the presence of an S residue at the N-terminus) able to inhibit DPP-IV was proposed.

## 1. Introduction

Food proteins include short amino-acid sequences, known as bioactive peptides, which show physiological functions and may modulate a variety of metabolic pathways resulting in health-promoting effects [1,2,3,4]. Bioactive peptides exhibit various physiological activities including anti-hypertensive effect, angiotensin-converting enzyme (ACE) and dipeptidyl-peptidase IV (DPP-IV) inhibitory activities, antioxidant, anti-inflammatory and immunomodulatory activities [5,6,7]. To date, milk proteins of various origins (such as bovine, goat, sheep, camel and buffalo) have been the ones most considered as precursors of bioactive peptides [8,9,10]. Nevertheless, some recent studies were designed for the identification of bioactive peptides derived from others animal proteins, especially from meat proteins of different species [7,11]. In recent years, the scientific community also exploited vegetable matrices as a source of bioactive peptides [5]. Legume and cereal proteins in particular have been studied for their ability to release bioactive peptides due to their worldwide diffusion as staple foods and their high protein content [12]. Common bean (*Phaseolus vulgaris*) contributes more than a third of the global legume production and is considered an important source of phytochemicals and bioactive peptides [13]. Previous studies identified ACE-inhibitory, DPP-IV-inhibitory, α-glucosidase-inhibitory, α-amylase-inhibitory and antioxidant peptides after hydrolysis or *in vitro* digestion of common bean proteins [14,15,16,17].

**Figure 1:**
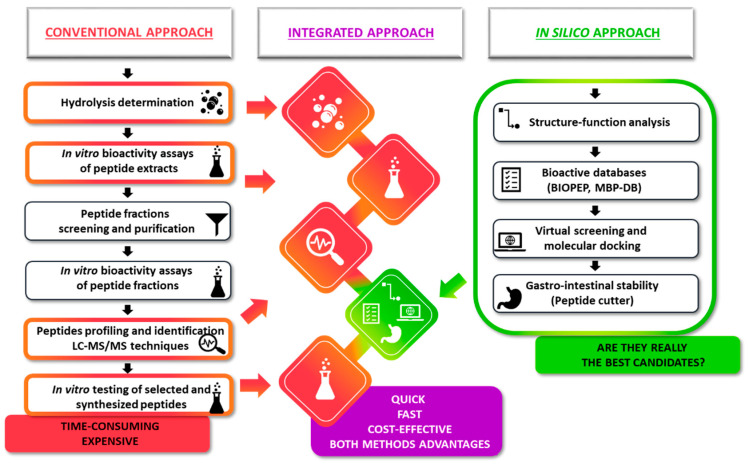
Schematic representation of conventional and *in silico* approaches in comparison with the new proposed integrated approach. The integrated approach combined the advantages of both the conventional and *in silico* approaches, using the pool of peptides identified in a real food hydrolysate as the starting point for subsequent application of selected bioinformatics tools.

Typically, the flow-chart for the identification of new bioactive peptides follows the so-called conventional or empirical approach (Figure 1).

The first step involves the hydrolysis of a selected substrate (through purified enzymes, fermentation or *in vitro* digestion) and the initial screening for a specific bioactivity. After which, the protein hydrolysate may be subjected to extensive bioassay-guided fractionation and purification procedures to separate peptides. Next, following the identification of the peptide(s) in the purified bioactive fraction by mass spectrometry, the synthetic putative bioactive peptide(s) is tested *in vitro* for biological activity validation [1,18]. This approach is tedious, time-consuming, expensive and not always successful. Nevertheless, the advantage of this approach is related to the identification of bioactive peptides actually present in the food (i.e., in fermented foods such as cheese) or in the food hydrolysates (i.e., hydrolysates used for the preparation of functional foods) or released after food digestion and therefore potentially active *in vivo* after the intake of the specific food.

More recently, bioinformatics-driven *in silico* approaches have been developed with the aim to provide a cost-effective and faster method for bioactive peptides discovery (Figure 1) [1,2,18]. These strategies take advantage of several bioinformatics tools including software for proteolysis prediction, software for molecular docking modelling (binding prediction with enzyme active site) and software for quantitative structure–activity relationship (QSAR) modelling. The application of the *in silico* strategy enables the virtual screening of a broad number of peptides. The selected candidates are further tested for *in vitro* validation of the postulated biological activity. This approach is rapid and cost-effective; however, it lacks an actual physiological significance since the real release of the bioactive peptide after hydrolysis or digestion has to be confirmed experimentally.

In our recent paper, we proposed a new integrated approach that combined peptidomics identification of the entire pool of peptides in a food hydrolysate with the application of several *in silico* analysis and structure–activity relationship modelling to identify novel bioactive peptides (Figure 1) [19]. The purpose was to combine the advantages of the conventional and *in silico* approaches using the pool of peptides identified in a real food hydrolysate as the starting point for subsequent application of selected bioinformatics tools. The first step in the proposed combined approach is the preparation of food hydrolysate. This can be achieved by using purified enzymes, microorganisms or through *in vitro* digestion [1,2,5]. Next, untargeted peptidomics is applied to identify all the peptides present in the sample [20]. The list obtained represents the pool of peptides that can be used for the subsequent *in silico* screening. The pool of peptides can also be obtained directly from foods such as fermented foods [19]. Biological activity assays can be carried out at this point on the hydrolysate. This step is optional but can be used as an initial bioactivity screening. The selection of putative novel bioactive peptides from the pool is then done by the application of chosen bioinformatics tools. First, the hundreds of identified peptides are screened with PeptideRanker software thus forming a list of dozens of potential bioactive peptides [21]. This software classifies the peptides based on a bioactivity prediction rank, without providing details on the type of activity [21]. Selected potential bioactive peptides are further screened using Pepsite2 software for the prediction of binding site interaction among them and the selected enzyme(s) [22]. Afterwards, the remaining potential bioactive peptides are analyzed to check if they have already been identified as bioactive peptides via software such as the BIOPEP database and the Milk Bioactive Peptides Database (MBPDB) [23,24]. The remaining peptides are subjected to structure–activity relationship modelling to select only those that have structural features similar to previously identified bioactive peptides. In the last step, the selected peptides can undergo *in silico* gastrointestinal digestion to simulate their stability under gastrointestinal conditions. This step is not required if the original pool of peptides has been obtained from an *in vitro*-digested sample. Finally, the *in vitro* validation of the supposed biological activity is carried out with synthetic peptides.

The described approach was applied in a previous study on Parmigiano Reggiano samples allowing the selection of 6 candidates to be tested *in vitro* starting from a pool of 415 peptides identified by peptidomics techniques [19]. Generally the selected peptides showed moderate activity but one of them (APFPE) was identified as a novel potent DPP-IV inhibitor.

The aim of this study was to further validate the described approach to identify new DPP-IV inhibitory peptides using a vegetable matrix (common bean) submitted to *in vitro* gastrointestinal digestion.

## 2. Materials and Methods

### 2.1. Materials

Enzymes and chemicals for the *in vitro* gastrointestinal digestion and for peptides quantification were from Sigma-Aldrich (Milan, Italy). Reagents for mass spectrometry analysis and SDS-PAGE electrophoresis were from Biorad (Hercules CA, USA.). Ultrafiltration units (Amicon Ultra-4 regenerated cellulose filters) with a molecular weight cut-off of 3 kDa were purchased from Millipore (Milan, Italy). *Phaseolus vulgaris* beans (pinto beans) were purchased from a local market (Reggio Emilia, Italy). All the other reagents were from Carlo Erba (Milan, Italy).

### 2.2. Preparation of the Pinto Bean Protein Extract

Pinto bean protein extract was prepared according to Carrasco-Castilla et al. [25]. Briefly, 100 g of dried beans were weighed and grounded to obtain a Pinto bean flour. The obtained flour was then defatted by extraction with hexane (600 mL) for 24 h at 4 °C under stirring. After hexane removal by rotary evaporation, the defatted flour was incubated with 75% acetone solution (375 mL of acetone + 125 mL of water) for 30 min at 4 °C to remove phenolic compounds. The suspension was then filtered through filter paper and the solid part was recovered. Proteins were further extracted by re-suspending the flour in 1000 mL of distilled water (1:10 w/v). The suspension was brought to pH 9.5 with NaOH 1 mol/L, stirred at 40 °C for 30 min and centrifuged at 5000× *g* for 20 min. The supernatant was then brought to pH 4.5 with HCl 1 mol/L to precipitate proteins. The protein precipitate was recovered by centrifugation at 10,000× *g* per 30 min. The obtained pellet was then lyophilized and stored at −20 °C. The amount of total proteins was determined by the Biuret assay. The protein yield was found to be more than 90%.

### 2.3. In Vitro Gastrointestinal Digestion

Pinto bean protein extract was *in vitro* digested according to the INFOGEST harmonized protocol [26]. In the oral step, 5 g of Pinto bean protein extract were mixed with 5 mL of simulated salivary fluid containing 150 U/mL of salivary α-amylase. After incubation for 5 min at 37 °C in a rotating wheel (10 rpm), 10 mL of simulated gastric fluid, containing 2000 U/mL of pepsin, were added and the gastric bolus was incubated for 2 h at 37 °C in a rotating wheel (10 rpm). For the intestinal, step 15 mL of intestinal fluid (added of bile salt and pancreatin, 200 U/mL based on trypsin activity) were added and the sample further incubated for 2 h at 37 °C in a rotating wheel (10 rpm). Aliquots were withdrawn during the different phases of the digestion procedure, immediately cooled in ice and stored at −80 °C for further analysis. The digestions were carried out in triplicate. Control digestions were performed by replacing pinto beans protein extract with water.

### 2.4. Assessment of Protein Hydrolysis during In Vitro Gastrointestinal Digestion

The hydrolysis of pinto bean protein extract was followed during *in vitro* gastrointestinal digestion by quantifying the amount of released free amino groups using the trinitrobenzensulfonic acid (TNBS) assay with leucine as standard [27]. The obtained data were corrected for the contribution of the control digestion.

### 2.5. Sodium Dodecyl Sulphate Poly-Acrylamide Gel Electrophoresis (SDS-PAGE)

SDS-PAGE electrophoresis was carried out as reported in Carrasco-Castilla et al. [25] on samples collected during the *in vitro* gastrointestinal digestion of pinto bean protein extract and control digestion. Samples were diluted in Laemmli buffer and boiled for 4 min to complete the denaturation process. Separating gel was prepared at 13% polyacrylamide concentration. An amount of 10 µL of each sample was loaded on the gel. Blue-StepTM Broad range marker (14–200 kDa) was utilized as a molecular weight standard. Proteins in the gel were detected by staining with Coomassie Blue.

### 2.6. Peptidomics Analysis of Low Molecular Weight Peptides by Nanoflow LC-ESI-QTOF-MS/MS

Low molecular weight peptides in samples withdrawn at the end of the *in vitro* gastrointestinal digestion were extracted by ultrafiltration with Amicon Ultra-4 nominal cut-off 3 kDa as previously described [28]. Nano LC/MS and tandem MS experiments were carried out on a 1200 Series Liquid Chromatographic two-dimensional system coupled with a 6520 Accurate-Mass Q-TOF LC/MS via a Chip Cube Interface (Agilent Technologies, Santa Clara, CA, USA). Chromatographic separation was performed on a ProtID-Chip-43 (II) including a 4 mm 40 nL enrichment column and a 43 mm × 75 μm analytical column, both packed with a Zorbax 300SB 5 μm C18 phase (Agilent Technologies, Santa Clara, CA, USA). The full description of the method is reported in Tagliazucchi et al. [15].

### 2.7. In Silico Analysis

#### 2.7.1. Identification of Previously Reported Bioactive Peptides

The pool of peptides identified in the in vitro-digested Pinto bean protein extract were searched against the BIOPEP database to identify peptides (100% sequence homology) with previously reported biological activities [23].

#### 2.7.2. Identification of Novel DPP-IV-Inhibitory Peptides

The *in silico* selection of novel DPP-IV-inhibitory peptides was carried out following the scheme reported in Martini et al. [19]. Briefly, the pool of peptides identified by peptidomics were firstly screened with Peptide Ranker software (v1, Shields Lab, Dublin, Ireland) to select only peptides with predicted potential biological activity [21]. Next, the probability of binding with the enzyme active site of selected peptides was analyzed by PepSite2 software (v2.3.01, Russell Lab, Oxford, UK) (PDB code of DPP-IV: 1NU6) [22]. Finally, the remaining peptides were subjected to structure–activity relationship modelling as described in Martini et al. [19].

Selected synthetic peptides (Bio-Fab Research, Rome, Italy) were then tested for their DPP-IV-inhibitory activity as reported in Tagliazucchi et al. [29]. Briefly, 145 μL of 0.1 mol/L Tris-HCl buffer (pH 7.0) were mixed with 10 μL of rat intestinal DPP-IV (0.1 U/mL in the assay) and 40 μL of synthetic peptides at different concentrations, in a 96-well plate. The reaction was started after 20 min of pre-incubation at 37 °C by the addition of 5 μL of 6.4 mmol/L glycine-proline-p-nitroanilide. After a further 20 min of reaction at 37 °C, the amount of released p-nitroanilide was measured by reading the absorbance at 405 nm in a microplate reader. Results were expressed as IC50 values (peptide concentration that inhibit the 50% of the enzymatic activity) by plotting the peptide concentration (base-10 logarithm) as a function of the enzyme inhibition percentage.

### 2.8. Statistical Analysis

All data are reported as mean ± standard deviation (SD) for three replicates for each prepared sample. GraphPad Prism 6.0 (GraphPad Software, San Diego, CA, USA) was used for univariate analysis of variance (ANOVA) with Tukey post-hoc test. The differences were considered significant when *p* < 0.05.

## 3. Results and Discussion

### 3.1. Assessment of Protein Hydrolysis during In Vitro Gastrointestinal Digestion

**Figure 2:**
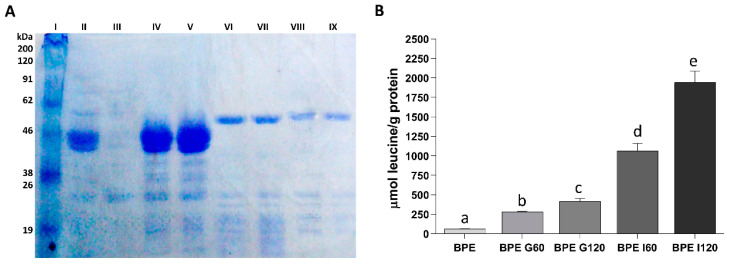
SDS-PAGE and degree of hydrolysis of bean proteins after the different steps of *in vitro* digestion. (**A**) SDS-PAGE of Pinto bean protein extract. Molecular weight marker is shown in lane 1. Protein pattern of Pinto bean protein extract is shown in lane 2. Sample after 60 min of gastric digestion is shown in lane 4. Sample after 120 min of gastric digestion is shown in lane 5. Sample after 60 min of intestinal digestion is shown in lane 6. Sample after 120 min of intestinal digestion is shown in lane 7. Samples after 60 and 120 min of control digestion with digestive enzymes but without bean proteins are shown in lane 8 and 9, respectively. (**B**) The amount of free amino groups released during the different phases of *in vitro* gastrointestinal digestion of Pinto bean protein extract. Results were expressed as μmol of leucine equivalent per g of the initial amount of proteins. Values represent means ± SD of triplicate digestions. Different letters indicate that the values are significantly different (*p* < 0.05). BPE: Pinto bean protein extract; BPE G60: Pinto bean protein extract after 60 min of gastric digestion; BPE G120: Pinto bean protein extract after 120 min of gastric digestion; BPE I60: Pinto bean protein extract after 60 min of intestinal digestion; BPE I120: Pinto bean protein extract after 120 min of intestinal digestion.

The hydrolysis of the Pinto bean protein extract during *in vitro* digestion was followed by both SDS-PAGE electrophoresis and free amino groups quantification. The electrophoretic profile of the Pinto bean protein extract before and during the *in vitro* digestion is reported in Figure 2A. The most visible bands in the Pinto bean protein extract (Figure 2A; Lane 2) were around 45 and 50 kDa and can be ascribed to the subunits α and β of phaseolins [15,30]. Additional bands, observed between 16 and 32 kDa, corresponded to proteins belonging to the phytohaemagglutinins [15,25]. The results suggested that phaseolins and phytohaemagglutinins were the major contributors to the protein profile of the Pinto bean protein extract. In vitro gastric digestion had little or no effect on phaseolins hydrolysis since the bands corresponding to these proteins were still visible after 60 min (Figure 2A; Lane 4) and at the end of the gastric digestion (Figure 2A; Lane 5). A previous study reported an insignificant effect of pepsin on the hydrolysis of purified phaseolins, especially when isolated from unheated beans [30]. However, the appearance of bands at low molecular weight may have indicated that some hydrolysis occurred also during the gastric phase of the digestion. After 60 and 120 min of intestinal digestion (Figure 2A; Lanes 6 and 7, respectively), a complete disappearance of the phaseolins and phytohaemagglutinins bands was observed indicating that Pinto bean proteins were abundantly degraded by intestinal proteases as, on the other hand, already suggested in other studies [15,25,30]. The bands visible in these samples corresponded to the digestive enzymes since the electrophoretic profile was identical to that of the control digestion performed without Pinto bean protein extract after 60 and 120 min of intestinal digestion (Figure 2A; Lanes 8 and 9, respectively).

The amount of free amino groups increased continuously during *in vitro* digestion (Figure 2B). After 60 min of gastric digestion the amount of free amino groups increased by about 4.7 times respect to the Pinto bean protein extract before digestion. Incubation for a further 60 min with the gastric fluid determined another 1.5 increase in the amount of free amino groups. The intestinal digestion determined a high increase in protein hydrolysis with an increment of 2.6 times after 60 min of intestinal digestion respect to the value recorded at the end of the gastric phase and a further increase of 1.8 in the next 60 min of intestinal digestion.

### 3.2. Peptidomics Profiles of Low-Molecular Weight Peptides after In Vitro Gastrointestinal Digestion and Identification of Previously Known Bioactive Peptides

Peptidomics analysis enabled the identification of 95 peptides after *in vitro* gastrointestinal digestion of Pinto bean protein extract. The mass spectrometry data and the sequence of all the identified peptides are reported in Table S1. The length of the amino acid sequences detected was between two and eight residues. Most of the identified peptides were di-peptides (38 peptides) and tri-peptides (29 peptides). A total of 30 peptides derived from the subunits α and β of phaseolin, which represent the 50% of common bean proteins [31]. Most of the identified di- and tri-peptides can be released from the hydrolysis of various proteins and most of them were anyway present in the phaseolin sequences.

The pool of peptides identified in the digested Pinto bean protein extract was analyzed with BIOPEP software to identify peptides with 100% sequence homology with known bioactive peptides. A total of 37 peptides previously identified as bioactive peptides were found in the database and are reported in Table 1.

The majority of the identified bioactive peptides were DPP-IV-inhibitors (17 peptides) and ACE-inhibitors (7 peptides), whereas 11 bioactive peptides showed both the inhibitory activities.

Several detected bioactive di-peptides were able to exert anti-hypertensive activity in spontaneously hypertensive rats (SHR). The di-peptide IY, isolated from a thermolysin digest of dried bonito, was able to decrease systolic blood pressure in SHR of 45 mmHg when administered intravenously at a dose of 10 mg/kg [32]. Similarly, the di-peptide LY, identified in an alcalase hydrolysate of rapeseed proteins, decreased systolic blood pressure in SHR of 26 mmHg after one single oral administration at a dose of 30 mg/kg [33]. Moreover, the dipeptide VF showed *in vitro* ACE-inhibitory activity (IC50 = 43.7 μmol/L), was able to decrease ACE activity in isolated rat aorta and, when tested in SHR at a dose of 5 mg/kg, decreased systolic blood pressure by 19 mmHg [34,35]. The dipeptides VF and IY have also been identified in human plasma after intake of a lactotripeptide-enriched milk beverage suggesting their absorption at the intestinal level [36,37].

### 3.3. Identification of Novel Potential DPP-IV-Inhibitory Peptides by In Silico Approach and Structure–Activity Relationship Modelling

The pool of peptides obtained from the *in vitro*-digested sample was submitted to the integrated procedure for the identification of novel potential DPP-IV inhibitory peptides as previously described [19]. As reported, this approach combines *in silico* analysis with structure–activity relationship modelling taking advantage of the pool of peptides identified by peptidomics technique.

We selected DPP-IV as a model enzyme to apply the integrated approach since, as reported in our previous work, the proposed approach works well when in-depth structure–activity relationship studies are present such as in the case of DPP-IV and inhibitory peptides [19,38,39]. The aminopeptidase DPP-IV is an intestinal brush-border prolyl-dipeptidase that hydrolyses incretins, a class of gastrointestinal hormones that stimulate the reduction of the hematic glucose level by fostering insulin secretion [40]. DPP-IV inhibitors are able to increase insulin secretion and, therefore, decrease blood glycaemia by preventing incretins inactivation [41]. Actually, the pharmacological treatment of type-2 diabetes with DPP-IV inhibitors is a therapeutic option that is becoming more and more accepted [41].

Firstly, the pool of peptides identified in the in vitro-digested sample was subjected to *in silico* screening by applying PeptideRanker and PepSite2 software. To get more information about the predictability of the potential bioactivity by the used software, we also included in the analysis the peptides with already reported DPP-IV-inhibitory activity. PeptideRanker is a software that allows the general prediction of peptide bioactivity without specifying the type of bioactivity [21]. Supplementary Table S2 reports the results about the PeptideRanker analysis for the 95 identified peptides. The authors suggested to consider all the peptides with a PeptideRanker threshold above 0.5 potentially bioactive [21]. However, since the 50% of the peptides with a rank in the range 0.4–0.5 have been already characterized as DPP-IV inhibitors, we decided to select for the further PepSite2 analysis all the peptides with a threshold above 0.4. As reported in Table S2, 21 peptides out of the 95 identified met this criterion. The selected 21 peptides were then subjected to PepSite2 analysis. This software is able to predict the probability that selected peptides could enter in the enzyme active site, expressed as *p*-value [22]. By selecting a threshold of 0.01 for the *p*-value, six peptides were then excluded from the following analysis (Table 2).

Of the remaining 15 potential DPP-IV-inhibitory peptides, nine of them had already been characterized as DPP-IV inhibitory peptides and, therefore, were excluded from the structure–activity relationship modelling. Peptides with high DPP-IV-inhibitory potency (IC50 < 100 μmol/L) possessed some typical structural features. Several studies suggested that the presence of a hydrophobic amino acid (I, L, V, A, F, and W) at the N-terminus and a P or A residue in second position were of paramount importance for the inhibitory activity [42,43,44]. A more recent structure–activity relationship study also suggested that for tri-peptides, in addition to the above reported structural features, the presence of a Q residue at the N-terminus and a hydrophobic amino acid (A, I, L, G, M, F) at the C-terminus were characteristics of peptides with IC50 value below 100 μmol/L [39]. Similarly, for tetra-peptides (or longer peptides) the presence of a P, L or R residue at the C-terminus and A, V, G or P in third position was observed in the most potent inhibitory peptides with a frequency higher than 10% [39]. Among the six selected peptides, three tetra-peptides met the structural characteristics of DPP-IV inhibitory peptides described above (Table 2). In particular, peptide FVPH, released from the α-subunit of phaseolin, possessed a F residue at the N-terminus and a P residue in third position. Peptide SIPR, released from acid β-fructofuranosidase, was characterized by the presence of a P residue in third position and a R residue at the C-terminus. Finally, the tetra-peptide SAPI, found in the sequence of phytohaemagglutinins and α-amylase inhibitor 2, displayed an A residue in second position and a P residue in third position. Therefore, these three tetra-peptides were selected for further *in vitro* validation of the DPP-IV-inhibitory activity.

### 3.4. In Vitro DPP-IV Inhibitory Activity of Synthetic Peptides

The three selected peptides were synthesized and assessed for their DPP-IV-inhibitory activity. All three were able to inhibit DPP-IV in the *in vitro* assay. Peptide SAPI was found to be the most powerful followed by SIPR and FVPH.

**Figure 3:**
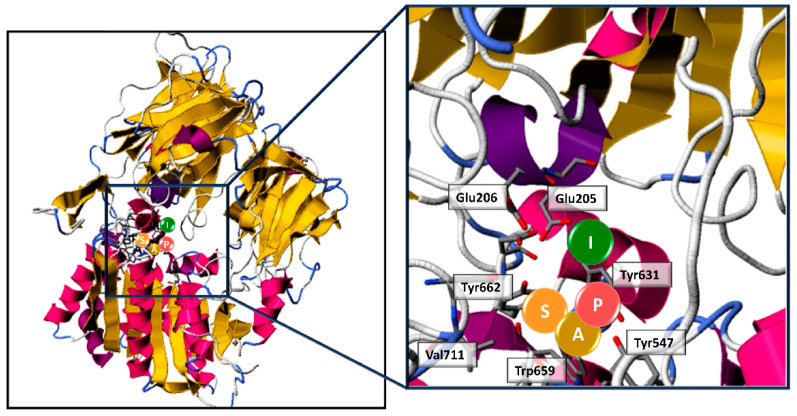
PepSite2 DPP-IV-SAPI binding modelling. Peptide binding site prediction of DPP-IV enzyme (PDB ID entry code: 1NU6) with SAPI. The insert shows the amino acids in the DPP-IV subsites S1 and S2 interacting with the tetra-peptides SAPI.

The IC50 value of the tetra-peptide SAPI was 57.7 ± 2.0 μmol/L, suggesting that it is a potent DPP-IV inhibitor [38,39,44]. The high inhibitory potency of SAPI was probably due to the combined presence of an A residue in second position and a P residue in third position. Indeed, it shared the C-terminal di-peptide sequence with the most potent DPP-IV-inhibitory peptide (IPI) identified to date [38,39,44]. The PepSite2 server was used to predict the possible interaction between SAPI and the DPP-IV binding sites by analyzing the enzyme active site residues closer to the peptide amino acids. As depicted in Figure 3, the peptide SAPI may have interacted with the DPP-IV active site, inhibiting the enzymatic activity. As reported by PepSite and illustrated in Figure 3, the peptide SAPI was positioned in the active site closer to key amino acids involved in the substrate-binding site and in the catalytic activity of DPPI-IV. This peptide was able to bind the residue Glu205 and Glu206 located at the S2 subsite as well as the aromatic amino acids Tyr547, Tyr631, Tyr662 and Trp659 that form part of the S1 pocket in the active site of DPP-IV [45]. Additional reported interaction were with the amino acids Val656, Asn710 and Val711 always located in the S1 subsite [45]. Finally, SAPI also interacted with the residue Ser630, which is part of the catalytic triad [45].

The tetra-peptide SIPR was found to be about 3.3 times less potent than SAPI with IC50 value of 189.0 ± 1.5 μmol/L. However, this peptide together with SAPI shared a S residue at the N-terminus. These results suggest the possible importance of the presence of a S residue at the N-terminus for DPP-IV inhibition, a feature never previously reported for DPP-IV-inhibitory peptides.

Finally, the peptide FVPH was found to be the less potent novel DPP-IV-inhibitory peptide identified in this study, showing an IC50 value of 480.6 ± 2.1 μmol/L. FVPH can be considered a multi-functional bioactive peptide. It has already been characterized as an antioxidant peptide released after hydrolysis of chickpea and fava bean [46,47].

## 4. Conclusions

In the current study, a previously developed integrated peptidomics and *in silico* approach was successfully applied to an in vitro-digested Pinto bean protein extract for the identification of new DPP-IV-inhibitory peptides. This combined approach allowed the selection of three new bioactive peptides whose inhibitory activity against DPP-IV was confirmed by *in vitro* testing with synthetic peptides. One identified peptide (SAPI) was characterized as one of the most potent inhibitory peptides described until now. Indeed, a new possible feature of DPP-IV-inhibitory peptides (i.e., the presence of a S residue at the N-terminus) was proposed.

This approach integrates laboratory techniques such as peptidomics analysis and *in vitro* bioactivity with bioinformatics analysis and structure–activity relationship modelling. The proposed method is cheap and fast but requires robust structure–activity relationship studies on the peptide features that confer inhibitory activity. However, the described procedure shortens the time of bioactive peptides discovery and costs of research. It combines the advantages of the conventional approach (i.e., detection of real peptides present in a hydrolysate) with that of pure *in silico* approach (i.e., prediction ability as well as economy and speed). Theoretically, it may be applied to every type of hydrolysates.

## Figures and Tables

**Table 1:** Peptides with previously reported biological activity identified in in vitro-digested Pinto bean protein extract.

Peptide Sequence	Protein Precursor	Bioactivity ^1^
AI	Various proteins	ACE-inhibition
AL	Various proteins	DPP-IV-inhibition
EI	Various proteins	ACE-inhibition DPP-IV-inhibition
EL	Various proteins	Antioxidant
EY	Various proteins	ACE-inhibition DPP-IV-inhibition
FVPH	α and β subunits of phaseolins	Antioxidant
GI	Various proteins	ACE-inhibition DPP-IV-inhibition
GL	Various proteins	ACE-inhibition DPP-IV-inhibition
IA	Various proteins	ACE-inhibition DPP-IV-inhibition
IE	Various proteins	ACE-inhibition
IH	Various proteins	DPP-IV-inhibition
II	Various proteins	DPP-IV-inhibition
IL	Various proteins	ACE-inhibition DPP-IV-inhibition
IP	Various proteins	ACE-inhibition DPP-IV-inhibition
IY	Various proteins	ACE-inhibition
LA	Various proteins	ACE-inhibition DPP-IV-inhibition
LH	Various proteins	DPP-IV-inhibition
LI	Various proteins	DPP-IV-inhibition
LKA	Various proteins	ACE-inhibition
LL	Various proteins	ACE-inhibition DPP-IV-inhibition
LP	Various proteins	DPP-IV-inhibition
LPQ	α and β subunits of phaseolins	DPP-IV-inhibition
LT	Various proteins	DPP-IV-inhibition
LY	Various proteins	ACE-inhibition
MI	Various proteins	DPP-IV-inhibition
ML	Various proteins	DPP-IV-inhibition
PR	Various proteins	ACE-inhibition
TI	Various proteins	DPP-IV-inhibition
TL	Various proteins	DPP-IV-inhibition
VAV	Various proteins	ACE-inhibition
VF	Various proteins	ACE-inhibition DPP-IV-inhibition
VI	Various proteins	DPP-IV-inhibition
VL	Various proteins	DPP-IV-inhibition
VM	Various proteins	DPP-IV-inhibition
VR	Various proteins	ACE-inhibition DPP-IV-inhibition
VV	Various proteins	DPP-IV-inhibition
YR	Various proteins	DPP-IV-inhibition

^1^ Potential bioactivities were achieved from the BIOPEP database. ACE: Angiotensin-converting enzyme; DPP-IV: Dipeptidyl peptidase IV.

**Table 2:** Selection of potential DPP-IV-inhibitory peptides as a function of PepSite2 *p*-value and structure–activity relationship (SAR) modelling. Selected peptides were reported in bold and underlined.

Sequence	PepSite2 *p*-Value for DPPIV (pdb: 1NU6)	SAR
		
PR	1.45 × 10^−4^	
IP *	1.98 × 10^−4^	
LP *	2.07 × 10^−4^	
LPQ *	4.91 × 10^−4^	
FT	1.49 × 10^−3^	
**SIPR**	1.77 × 10^−3^	R at the C-terminus and *p* in penultimate position
GI *	2.07 × 10^−3^	
MI *	2.12 × 10^−3^	
**SAPI**	2.23 × 10^−3^	*p* in penultimate position and A in second position
ML *	2.25 × 10^−3^	
GL *	3.53 × 10^−3^	
**FVPH**	3.56 × 10^−3^	F at the N-terminus and *p* in penultimate position
VF *	3.63 × 10^−3^	
FV	3.63 × 10^−3^	
AL *	5.87 × 10^−3^	

* Previously identified as DPPIV-inhibitor. Threshold was set at 0.01. Peptides with *p*-value below the threshold are not shown.

## Data Availability

The data presented in this study are available in here and in supplementary material.

## Supplementary Materials

The following are available online at https://www.mdpi.com/article/10.3390/cimb44010011/s1. Table S1: Peptides identified in the <3 kDa permeate obtained from Pinto bean protein extract after standardized *in vitro* gastrointestinal digestion. Table S2: Potential bioactive peptides (threshold > 0.4) identified through PeptideRanker software. Peptides are sorted in decreasing peptide rank values. Threshold was set at 0.4.
