# Supplementary material for: Application of a Combined Peptidomics and In Silico Approach for the Identification of Novel Dipeptidyl Peptidase-IV-Inhibitory Peptides in In Vitro Digested Pinto Bean Protein Extract
Source: Curr Issues Mol Biol. 2021 Dec 28;44(1):139–51. doi: 10.3390/cimb44010011 (PMC8929122; doi:10.3390/cimb44010011)
Supplement: Supplementary file 1 [file cimb-44-00011-s001.zip › Table S1.pdf]

Table S1. Peptides identified in the <3 kDa permeate obtained from Pinto bean protein extract after standardized *in vitro* gastro-intestinal digestion.

Sequence	*Observed [M+H]+a*	*Calculated [M+H]+a*	*Error (ppm)*	*Charge*	*Protein[b]*	*Fragment*
VVNEGEA	717.3438	717.3414	4.74	+1	α and β subunits of phaseolins	f(295-301)<br>f(289-295)
IDSEQ	591.2610	591.2620	1.91	+1	α and β subunits of phaseolins	f(225-229)<br>f(219-223)
EEIN	504.2322	504.2300	4.36	+1	α and β subunits of phaseolins	f(200-203)<br>f(199-202)
ALDGK	503.2820	503.2824	-0.79	+1	α and β subunits of phaseolins	f(377-381)<br>f(371-375)
VYPS	465.2343	465.2344	-0.21	+1	Phytohemagglutinins	f(199-202)
IPAGT	458.2621	458.2609	2.62	+1	α and β subunits of phaseolins	f(137-141)<br>f(136-140)
TDNV	448.2059	448.2038	4.69	+1	α and β subunits of phaseolins	f(367-370)<br>f(361-364)
VIVN	444.2826	444.2817	2.03	+1	α and β subunits of phaseolins	f(221-224)<br>f(215-218)
FVDG	437.2014	437.2031	-3.89	+1	α subunit of phaseolins	f(407-410)
LDGK	432.2436	432.2453	-3.93	+1	α and β subunits of phaseolins	f(378-381)<br>f(372-375)
KSLS	434.2627	434.2609	4.14	+1	β subunit of phaseolins	f(237-240)
VVNEGEAH	427.7058	427.7038	4.68	+2	α and β subunits of phaseolins	f(295-302)<br>f(289-296)
FEE	424.1734	424.1714	4.72	+1	Various protein	/
DVLG	403.2194	403.2187	1.74	+1	α and β subunits of phaseolins	f(382-385)<br>f(376-379)
VIPA	399.2609	399.2602	1.75	+1	α and β subunits of phaseolins	f(331-334)<br>f(325-328)
LEH	398.2049	398.2034	3.77	+1	Various protein	/
IEH	398.2049	398.2034	3.77	+1	Various protein	/
SAPI	387.2242	387.2238	1.03	+1	Phytohemagglutinins	f(73-76)
HVE	384.1863	384.1878	-3.90	+1	α and β subunits of phaseolins	f(302-304)<br>f(296-298)
ALDG	375.1880	375.1874	1.60	+1	α and β subunits of phaseolins	f(377-380)<br>f(371-374)
ILE	374.2295	374.2286	2.40	+1	α subunit of phaseolins	f(190-192)
IIQ	373.2450	373.2445	1.34	+1	α and β subunits of phaseolins	f(156-158)<br>f(155-157)

ILQ	373.2450	373.2445	1.34	+1	Phytohemagglutinins	f(38-40)
NNPQIH	361.6826	361.6826	0.00	+2	α and β subunits of phaseolins	f(164-169) f(163-168)
LPQ	357.2143	357.2132	3.08	+1	α and β subunits of phaseolins	f(90-92) f(90-92)
SIE	348.1782	348.1765	4.88	+1	Various protein	/
SLE	348.1782	348.1765	4.88	+1	Various protein	/
EIS	348.1763	348.1765	-0.57	+1	Various protein	/
EIS	348.1763	348.1765	-0.57	+1	Various protein	/
VVE	346.1989	346.1973	4.62	+1	Various protein	/
LVN	345.2132	345.2132	0.00	+1	Various protein	/
IVN	345.2132	345.2132	0.00	+1	Various protein	/
LVKPDD	343.6909	343.6896	3.78	+2	α and β subunits of phaseolins	f(111-116) f(111-116)
YR	338.1813	338.1823	-2.96	+1	Various protein	/
VNPDPK	335.1835	335.1819	4.77	+2	α and β subunits of phaseolins	f(146-151) f(145-150)
DGK	319.1610	319.1612	-0.62	+1	α and β subunits of phaseolins	f(379-381) f(373-375)
VVT	318.2034	318.2023	3.46	+1	Various protein	/
EY	311.1252	311.1238	4.50	+1	Various protein	/
ISS	306.1649	306.1660	-3.59	+1	Various protein	/
LSS	306.1649	306.1660	-3.59	+1	Various protein	/
AIV	302.2080	302.2074	1.99	+1	α and β subunits of phaseolins	f(290-292) f(284-286)
LY	295.1640	295.1652	-4.07	+1	Various protein	/
IY	295.1640	295.1652	-4.07	+1	Various protein	/
FVDAH	294.6440	294.6426	4.75	+2	β subunit of phaseolins	f(401-405)
GIT	290.1714	290.1710	1.38	+1	Various protein	/
GLT	290.1714	290.1710	1.38	+1	Various protein	/
VAV	288.1932	288.1918	4.86	+1	Various protein	/
LVG	288.1925	288.1918	2.43	+1	α and β subunits of phaseolins	f(305-307) f(299-301)

FVDGH	287.6360	287.6346	4.87	+2	α subunit of phaseolins	f(407-411)
SKPET	281.1479	281.1476	1.07	+2	α and β subunits of phaseolins	f(83-87)
VR	274.1884	274.1874	3.65	+1	Various protein	/
PR	272.1704	272.1717	-4.78	+1	Various protein	/
IH	269.1610	269.1608	0.74	+1	Various protein	/
LH	269.1610	269.1608	0.74	+1	Various protein	/
FT	267.1330	267.1339	-3.37	+1	Various protein	/
RIIQ	265.1771	265.1765	2.26	+2	α and β subunits of phaseolins	f(155-158) f(154-157)
VF	265.1534	265.1547	-4.90	+1	Various protein	/
FV	265.1547	265.1547	0.00	+1	Various protein	/
MI	263.1429	263.1424	1.90	+1	Various protein	/
ML	263.1429	263.1424	1.90	+1	Various protein	/
EI	261.1448	261.1445	1.15	+1	Various protein	/
EL	261.1448	261.1445	1.15	+1	Various protein	/
IE	261.1448	261.1445	1.15	+1	Various protein	/
LE	261.1448	261.1445	1.15	+1	Various protein	/
FVPH	250.1368	250.1368	0.00	+2	α and β subunits of phaseolins	f(282-285) f(276-279)
VM	249.1279	249.1267	4.82	+1	Various protein	/
DI	247.1300	247.1288	4.86	+1	Various protein	/
DL	247.1300	247.1288	4.86	+1	Various protein	/
II	245.1862	245.1860	0.82	+1	Various protein	/
IL	245.1862	245.1860	0.82	+1	Various protein	/
LI	245.1862	245.1860	0.82	+1	Various protein	/
LL	245.1862	245.1860	0.82	+1	Various protein	/
KLIN	244.1656	244.1656	0.00	+2	α and β subunits of phaseolins	f(397-400) f(391-394)
SIPR	236.6481	236.6475	2.45	+2	Acid β-fructofuranosidase	f(421-424)

TL	233.1503	233.1496	3.00	+1	Various protein	/
TI	233.1503	233.1496	3.00	+1	Various protein	/
LT	233.1488	233.1496	-3.43	+1	Various protein	/
IT	233.1488	233.1496	-3.43	+1	Various protein	/
VL	231.1699	231.1703	-1.73	+1	Various protein	/
VI	231.1699	231.1703	-1.73	+1	Various protein	/
LP	229.1549	2291547	0.87	+1	Various protein	/
IP	229.1549	2291547	0.87	+1	Various protein	/
VAPR	221.6429	221.6423	2.71	+2	Phosphoribosylaminoimidazole carboxylase	f(265-268)
VV	217.1550	217.1547	1.38	+1	Various protein	/
LA	203.1394	203.1390	1.97	+1	Various protein	/
IA	203.1394	203.1390	1.97	+1	Various protein	/
AL	203.1394	203.1390	1.97	+1	Various protein	/
AI	203.1394	203.1390	1.97	+1	Various protein	/
LKE	195.1231	195.1234	-1.54	+2	Various protein	/
IKE	195.1231	195.1234	-1.54	+2	Various protein	/
GL	189.1237	189.1234	1.59	+1	Various protein	/
GI	189.1237	189.1234	1.59	+1	Various protein	/
VPH	176.6025	176.6026	-0.57	+2	α and β subunits of phaseolins	f(283-285) f(277-279)
LKA	166.1211	166.1206	3.01	+2	Various protein	/
IKA	166.1211	166.1206	3.01	+2	Various protein	/
