# Supplementary material for: Application of a Combined Peptidomics and In Silico Approach for the Identification of Novel Dipeptidyl Peptidase-IV-Inhibitory Peptides in In Vitro Digested Pinto Bean Protein Extract
Source: Curr Issues Mol Biol. 2021 Dec 28;44(1):139–51. doi: 10.3390/cimb44010011 (PMC8929122; doi:10.3390/cimb44010011)
Supplement: Supplementary file 1 [file cimb-44-00011-s001.zip › Table S2.pdf]

**Table S2**. Potential bioactive peptides (threshold > 0.4) identified through PeptideRanker software. Peptides are sorted in decreasing peptide rank values. Threshold was set at 0.4.

| ***Peptide rank value*** | ***Sequence*** |
|---|---|
| 0.89 | ML |
| 0.82 | FT |
| 0.81 | GL |
| 0.81 | VF |
| 0.80 | LP |
| 0.79 | PR |
| 0.77 | FV |
| 0.70 | MI |
| 0.67 | FVPH |
| 0.62 | LL |
| 0.58 | IP |
| 0.57 | SIPR |
| 0.53 | YR |
| 0.52 | GI |
| 0.52 | LY |
| 0.47 | FVDG |
| 0.44 | FVDGH |
| 0.44 | AL |
| 0.43 | LPQ |
| 0.42 | SAPI |
| 0.40 | IL |
| 0.34 | LI |
| 0.34 | LH |
| 0.34 | VNPDPK |
| 0.33 | VM |
| 0.33 | FVDAH |
| 0.33 | DL |
| 0.33 | GLT |
| 0.32 | IY |
| 0.31 | ALDG |
| 0.31 | LA |
| 0.31 | IPAGT |
| 0.30 | NNPQIH |
| 0.28 | VAPR |
| 0.23 | ALDGK |

| | |
|---|---|
| 0.23 | GIT |
| 0.23 | VPH |
| 0.22 | LDGK |
| 0.22 | II |
| 0.22 | VIPA |
| 0.21 | IH |
| 0.21 | DGK |
| 0.20 | AI |
| 0.2 | LVG |
| 0.20 | VYPS |
| 0.19 | DVLG |
| 0.18 | ILQ |
| 0.18 | IA |
| 0.16 | FEE |
| 0.15 | RIIQ |
| 0.15 | IIQ |
| 0.14 | TL |
| 0.14 | DI |
| 0.14 | LKA |
| 0.13 | VL |
| 0.13 | LSS |
| 0.13 | KLIN |
| 0.11 | SKPET |
| 0.11 | LT |
| 0.11 | ISS |
| 0.11 | VR |
| 0.11 | IKA |
| 0.11 | LVKPDD |
| 0.10 | AIV |
| 0.10 | ILE |
| 0.09 | SLE |
| 0.08 | IT |
| 0.08 | LVN |
| 0.08 | SIE |
| 0.08 | LEH |
| 0.08 | IEH |
| 0.07 | TI |
| 0.07 | EL |
| 0.07 | EY |
| 0.07 | IVN |

| | |
|---|---|
| 0.06 | VI |
| 0.06 | LE |
| 0.06 | ELS |
| 0.06 | IDSEQ |
| 0.05 | EI |
| 0.05 | IE |
| 0.05 | EIS |
| 0.05 | LKE |
| 0.05 | VIVN |
| 0.05 | VVNEGEAH |
| 0.04 | TDNV |
| 0.04 | VVNEGEA |
| 0.04 | KSLS |
| 0.04 | VAV |
| 0.04 | IKE |
| 0.04 | EEIN |
| 0.04 | HVE |
| 0.03 | VV |
| 0.03 | VVT |
| 0.02 | VVE |
